# Prophylactic interventions for preventing macular edema after cataract surgery in patients with diabetes: A Bayesian network meta-analysis of randomized controlled trials

**DOI:** 10.1016/j.eclinm.2022.101463

**Published:** 2022-05-20

**Authors:** Ruiheng Zhang, Li Dong, Qiong Yang, Yueming Liu, Heyan Li, Wenda Zhou, Haotian Wu, Yifan Li, Yitong Li, Chuyao Yu, Wenbin Wei

**Affiliations:** Beijing Tongren Eye Center, Beijing Key Laboratory of Intraocular Tumor Diagnosis and Treatment, Beijing Ophthalmology & Visual Sciences Key Lab, Medical Artificial Intelligence Research and Verification Key Laboratory of the Ministry of Industry and Information Technology, Beijing Tongren Hospital, Capital Medical University, Beijing, China

**Keywords:** Diabetes, Macular edema, Cataract surgery, NSAIDs, Anti-vascular endothelial growth factor, PME, Postoperative macular edema, BCVA, Best-corrected visual acuity, anti-VEGF, Anti-vascular endothelial growth factor injection, NSAIDs, Nonsteroidal anti-inflammatory drugs, CI, Confidence interval, DR, Diabetic retinopathy, DME, Diabetic macular edema, VEGF, Vascular endothelial growth factor, PRISMA, Preferred Reporting Items for Systematic Reviews and Meta-Analyses, LogMAR, Logarithm of the Minimum Angle of Resolution, OR, Odds ratios, MD, Mean difference, IDI, Intravitreal dexamethasone implant

## Abstract

**Background:**

Diabetes significantly increases the risk of postoperative macular edema (PME) after cataract surgery, leading to potential worst post-operative outcomes. This study aims to compare the effect of different prophylactic interventions in improving postoperative anatomic and visual acuity outcomes of diabetes patients who underwent cataract surgery.

**Methods:**

We searched MEDLINE, Embase, Web of Science databases from inception until February 2nd, 2022, for studies including studies reporting PME events and/or best-corrected visual acuity (BCVA) outcomes. Random-effects Bayesian network meta-analysis was performed to compare the efficiency of intravitreal anti-vascular endothelial growth factor injections (anti-VEGF), nonsteroidal anti-inflammatory drugs (NSAIDs) and topical steroids eye drop at 1 week, 1 month, 3 months, 6 months after cataract surgery.

**Findings:**

The total of 2566 participants from 17 randomized controlled trials were included in the network meta-analysis, with moderate risk of bias and no evidence of publication of bias. Compared to placebo/steroid eye drop alone, patients received additional topical NSAIDs or intravitreal anti-VEGF injections had lower risk of PME at 1 month (NSAIDs: OR=0·221, 95% Confidence interval [CI], 0·044–0·755, *I^2^*=0·0%, 5 studies; anti-VEGF: OR=0·151, 95%CI, 0·037–0·413, *I^2^*=0·0%, 5 studies) and 3 month (NSAIDs: OR=0·370, 95%CI, 0·140–0·875, *I^2^*=0·0%, 8 studies; anti-VEGF: OR=0·203, 95%CI, 0·101–0·353, *I^2^*=0·0%, 4 studies) after cataract surgery. Further, additional anti-VEGF exhibited better BCVA outcome at 1 month (mean difference of LogMAR: -0·083, 95%CI, -0·17 to -0·014, *I^2^*=62·0%, 5 studies), and 3 months (mean difference of LogMAR: -0·061, 95%CI, -0·11 to -0·011, *I^2^*=0·0%, 5 studies) after cataract surgery. Such additional benefits did not reach statistic significant at 6 months after surgery.

**Interpretation:**

Our data suggests that compared to placebo/steroid eye drop alone, additional prophylactic anti-VEGF intervention could be considered for preventing the occurrence of PME after cataract surgery in patients with diabetes.

**Funding:**

Research and Development of Special (2020-1-2052); Science & Technology Project of Beijing Municipal Science & Technology Commission (Z201100005520045, Z181100001818003).

**Research in context**

**Evidence before the study**

Diabetes significantly increases the risk of postoperative macular edema (PME), leading to poor postoperative visual acuity, yet it is still inconclusive which prophylactic intervention should be used during the perioperative period. Literature search for this network meta-analysis was performed using MEDLINE, Embase, Web of Science, and ClinicalTrials.gov databases on August 4, 2021, and updated on February 8, 2022, using variants and combinations of search terms relating to macular edema and cataract surgery. Randomized controlled clinical trials that compared different prophylactic interventions for preventing PME after cataract surgery in diabetes patients were reviewed, which suggested that non-steroidal anti-inflammatory drugs (NSAIDs) eye drop, intravitreal anti-VEGF, and steroid agents are potentially effective in preventing PME and improving postoperative best-corrected visual acuity (BCVA). However, because the interpretation of this evidence is limited by small sample size and the lack of direct comparisons between intravitreal anti-VEGF and NSAIDs, a network meta-analysis is needed that compares the effect of different prophylactic interventions for preventing PME in diabetes patients after cataract surgery, especially regarding BVCA outcomes.

**Added value of the study**

Through the extensive literature search, 17 studies were eligible for this network meta-analysis and exhibited a low to moderate risk of bias and no evidence of publication bias. Based on 17 randomized controlled trials with 2566 diabetes participants, additional topical NSAIDs and intravitreal anti-VEGF injection show significantly lower risk of PME compared to placebo/steroid eye drop alone. This study further highlights that additional anti-VEGF brought better BCVA outcome after cataract surgery.

**Implications of all the available evidence**

Additional intravitreal anti-VEGF could prevent the occurrence of PME and improves postoperative BCVA, and can be integrated into the clinical practice for managing diabetes patients with cataract. In the future, head-to-head clinical trials are needed to validate this synthesized evidence. Furthermore, more prospective trials are needed to investigate the effect of intravitreal longer-lasting steroids on PME and BCVA outcomes in diabetes patients undergoing cataract surgery.

Alt-text: Unlabelled box

## Introduction

The number of adults with diabetes was expected to surpass 700 million globally by 2045.^1^ Diabetes is one of the major risk factors of cataract.^2^ According to Wisconsin Epidemiologic Study of Diabetic Retinopathy, 8·3% younger-onset and 24·9% older-onset diabetes would undergo cataract surgery.^3^ Diabetes also significantly increases the risk of postoperative macular edema (PME), leading to poor postoperative visual acuity. By analyzing 81,984 eyes undergoing cataract surgery, evidence has been found that 2·15% diabetes patients without diabetic retinopathy (DR) developed PME. The incidence rates of PME would increase to 9·43%, 9·75%, 7·69%, and 12·07% if patients were complicated with mild, moderate, severe non-proliferate DR and proliferate DR, respectively.^4^ Unfortunately, compared to non-diabetes patients, diabetes patients who develop PME has limited visual recovery at 3 months after surgery.^5^

PME in the diabetes may contribute to two different mechanisms: susceptible condition of diabetic macular edema (DME), or the unset of pseudophakic cystoid macular edema.^6^^,^^7^ Accordingly, several perioperative interventions have been proposed to reduce the incidence rate of PME in diabetes underwent cataract surgery. Nonsteroidal anti-inflammatory drugs (NSAIDs) inhibit prostaglandins synthesis during surgical inflammatory response.^6^ Thus, NSAIDs is used as one of the common perioperative interventions in preventing PME.^8^ Recently, evidence has found diabetic patients who underwent cataract surgery might also benefit from NSAIDs.^9^^,^^10^ Because vascular endothelial growth factor (VEGF) is the primary factor for retinal vascular hyperpermeability during diabetic retinopathy.^11^ Anti-VEGF is now used as the primary therapy for DME, effectively eliminating macular edema and improving vision in most DME patients.^12^ Off-label treatment with Anti-VEGF showed to be effective in preventing PME.^13^ There are also some evidence that found multiple inflammatory mediators involves the pathogenesis of DME, which gives the rationales for applying steroids agents as perioperative interventions.^14^

Prophylactic NSAIDs eye drop usually requires 2–4 times/day for 1 month duration, according to a recent trial.^9^ In contrast, anti-VEGF therapy only requires single injection during surgery, but with much more expense.^15^ For now, there was a lack of high-quality evidence on the effects of prophylactic interventions for preventing macular edema after cataract surgery in patients with diabetes, especially regarding best-corrected visual acuity (BCVA) outcome.^16^^,^^17^ Thus, in this study, we performed a systematic review and network meta-analysis intending to derive evidence-based clinical guidelines for PME prevention in diabetic patients.

## Method

### Search strategy

This study followed the Preferred Reporting Items for Systematic Reviews and Meta-Analyses (PRISMA) checklist extension statement for network meta-analysis guideline.^18^ A systematic search was performed by two authors (Zhang, Dong) independently on 30 July 2021. We searched MEDLINE (published between 1946 and 30 July 2021), Embase (published 1974 to 30 July 2021), Web of Science (1975 to 30 July 2021). Details for search strategy were listed in Supplementary material (Appendix 1). Relevant articles from the reference lists of the retrieved articles were also searched. Search results were restricted to human study and English language only. The search was updated on February 8, 2022.

### Inclusion and exclusion criteria

Two authors (Zhang and Dong) independently reviewed all studies by title and abstract. After primary selection, two authors (Zhang, Dong) independently screened full-text studies, and considered for inclusion if they met the following criteria: (1) Randomized controlled trails; (2) Including diabetes patients without DME and undergoing cataract surgery; (3) With two or more interventions in preventing PME, including but not limited to anti-VEGF (Bevacizumab, Ranibizumab and Aflibercept), NSAIDs (Nepafenac, Ketorolac, Bromfenac, and Diclofenac), topical steroids (Betamethasone, Dexamethasone, and Prednisolone); and (4) Reporting PME events and/or BCVA outcomes. We excluded studies with the following criteria:(1) With insufficient data for methodological quality assessment; (2) single-arm trials; (3) Reviews, editorials, letters, abstracts, case reports, or practice guidelines. Any disagreements about study inclusion/exclusion that could not be resolved by discussion between two authors (Zhang and Dong) were decided by a third author (Wei).

### Risk of bias assessment

Study quality was assessed by revised Cochrane risk-of-bias tool for randomized trials (RoB 2).^19^ The methodology examined the following aspects of each trial: bias arising (1) from the randomization process; (2) deviations from intended interventions; (3) missing outcome data; (4) measurement of the outcome; (5) selection of the reported result. These answers lead to judgments of “low risk of bias,” “some concerns,” or “high risk of bias”.

### Data extraction

Two authors (Zhang and Dong) independently extracted essential characteristics of included studies, including diabetic retinopathy status, longest follow-up duration, routine perioperative intervention for all participants, and mean baseline central subfield thickness. We further extracted PME and BCVA (Logarithm of the Minimum Angle of Resolution, LogMAR) outcomes reported from included studies. Macular edema that developed in diabetic patients often has mixed characteristics of DME and pseudophakic cystoid macular edema.^20^ Thus, PME was defined as macular edema with all kinds of morphologic features. Missing data were read from figures by using GetData Graph Digitizer 2·26 (http://getdata-graphdigitizer.com). For BCVA outcomes, we calculated BCVA improvement from baseline by Epicalc (Version 1·0·2, Brixton Health). BCVA recorded as ETDRS letters were transformed to LogMAR.^21^

### Statistical analysis

All statistical analysis was performed using R Statistical Software (version 4·1·1; R Foundation for Statistical Computing, Vienna, Austria), Stata (17·0, StataCorp LLC, College Station, TX). We performed a random-effects network meta-analysis within a Bayesian setting by using ‘Gemtc’ package for R (Gemtc version 1·0–1, Repository CRAN). Continuous variables were modeled using mean difference (MD), whereas binary outcomes were modeled using a binomial likelihood and logit link. We utilized Markov chain Monte Carlo methods to estimate pooled Odds ratios (ORs) and MD with 95% confidence intervals (CI). To check convergence, we used the Gelman and Rubin diagnostic plot.^22^ Eventually, Markov chain Monte Carlo simulation was set on four parallel chains, 5000 burn-in iterations and 100,000 actual simulation iterations. We used the nodesplit method to evaluate the consistency of our network model.^23^ We ranked treatments based on the analysis of surface under the cumulative ranking.^24^ Direct pair-wise meta-analysis was used a random effects model to assessed heterogeneity with the *I^2^* statistic. According to Higgins and Thompson,^25^ heterogeneity was assessed by the *I^2^* statistic values: ∼25% represented low heterogeneity; ∼50% represented medium heterogeneity; and ∼75% represented high heterogeneity. The comparison-adjusted funnel plot was used to assess publication bias and small-study effects for outcomes including at least 10 studies.^26^ All the included studies in this review had received ethical approval prior to data collection.

### Role of the funders

The funders had no role in the study design, data collection, data analysis, data interpretation, or in the writing of the manuscript. The corresponding author (WBW), RHZ and LD have full access to all data in the study and they took the decision to submit the manuscript for publication.

## Results

### Literature search

**Figure 1:**
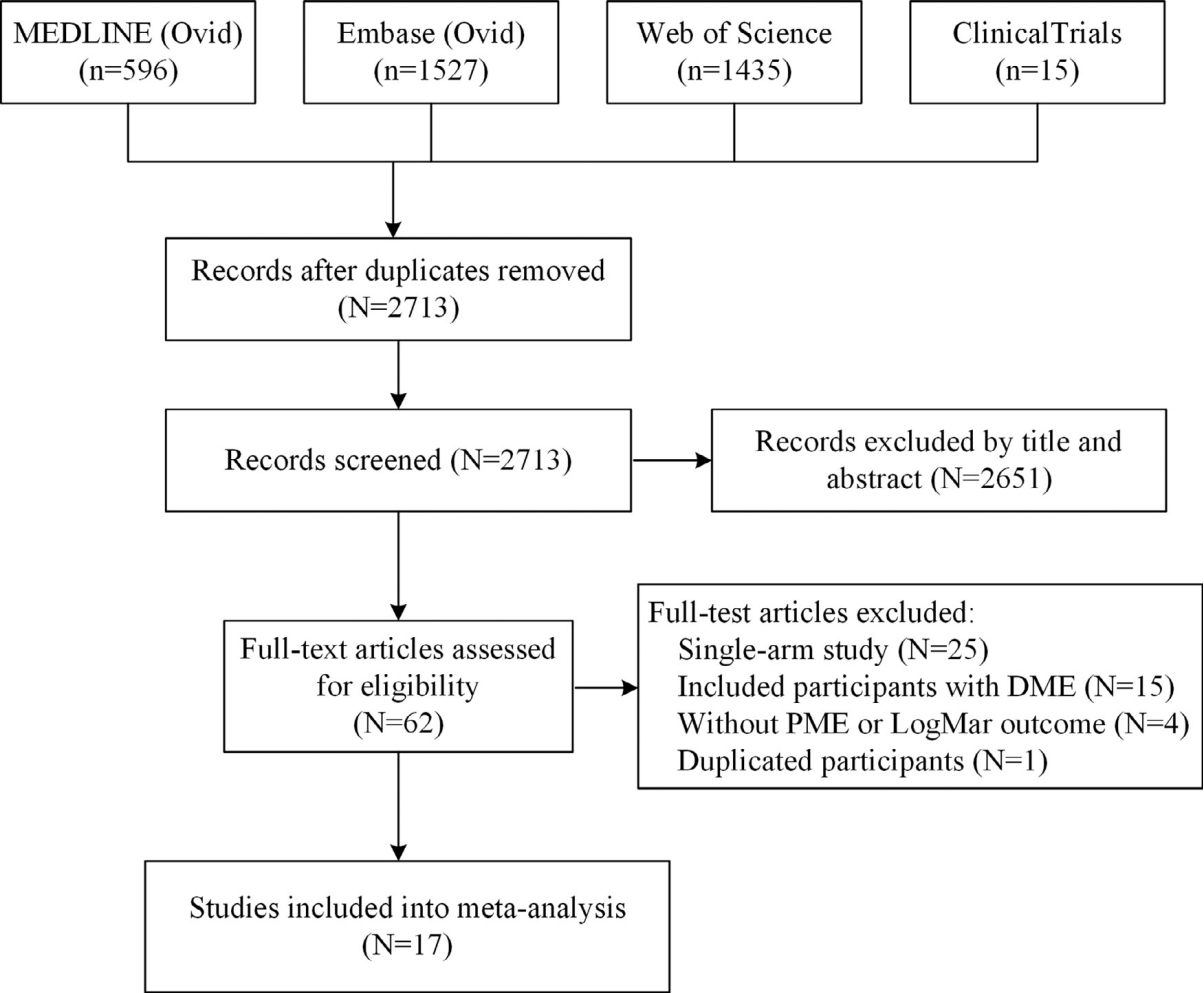
Flowgram of included study. DME, diabetic macular edema; PME, postsurgical macular edema; LogMar, Logarithm of the Minimum angle of resolution.

**Figure 2:**
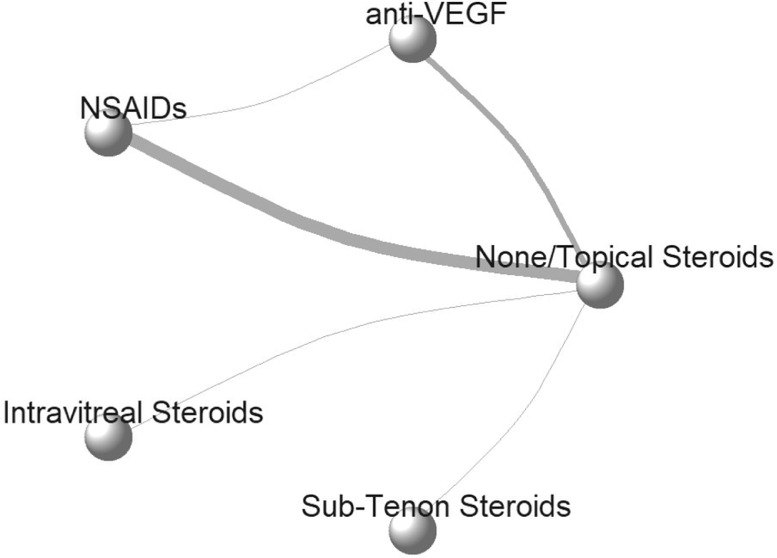
Network plots for diabetic macular edema and best corrected visual acuity. anti-VEGF, anti-vascular endothelial growth factor therapy; NSAIDs, Nonsteroidal anti-inflammatory drugs. Each circle represents one intervention, and the thickness of connected lines indicate number of trials for each comparison.

Through systemic research, we identified 2713 unique studies. After reviewing the titles and abstracts of these articles, a further 2651 were excluded. Among the remaining 62 full-text studies, 17 of them into final network meta-analysis (Figure 1). In all, a total of 2566 participants from 17 randomized controlled trials were included in the finally network meta-analysis (Table 1 and Supplementary Table 1).^9^^,^^10^^,^^13^^,^^14^^,^^27^, ^28^, ^29^, ^30^, ^31^, ^32^, ^33^, ^34^, ^35^, ^36^, ^37^, ^38^, ^39^ 17 studies reported 5 perioperative interventions: topical steroid eye drop, topical NSAIDs eye drop, intravitreal anti-VEGF agents injection, intravitreal steroid injection, and sub-tenon steroid injection. All studies used no intervention or topical steroid eye drop alone (None/Topical Steroids) as reference treatments. Thus, we performed this network meta-analysis by comparing the effect of None/topical steroid eye drop alone with additional these 4 kinds of interventions (Figure 2).

**Table 1:** Characteristics of included studies.

Study (Author, year)	Country	Participants	Follow-up, month	Routine Perioperative intervention	Definition of PME	Intervention	Number of Eyes	Mean Age, year (SD)	Mean baseline CST (SD)
Ahmadabadi 2010	Iran	T2D and moderate NPDR	6 months	0·1% betamethasone	Based on OCT and FA	None	21	62 (11)	188 (24)
						IV-TA (2 mg)	20	63 (11)	197 (27)
Alnagdy 2018	Egypt	DM without DR	3 months	Steroid eye drop	CST changes >40 μm	Artificial tear	40	58 (9)	226 (15)
						0·1% Nepafenac	20	58 (10)	232 (18)
						0·4% Ketorolac	20	63 (8)	228 (21)
Chae 2014	South Korea	DM with DR	6 months	Not mentioned	CST changes >30% increase	Sham	37	67 (8)	253 (36)
						IV-R (0·5 mg)	39	63 (9)	256 (27)
Elsawy 2013	Egypt	DM with DR	12 months	0·1% dexamethasone	Based on OCT and FA	None	35	NA	257 (17)
						Ketorolac 0·4%	35	NA	258 (17)
Endo 2010	Japan	DM with and without DR	6 months	None	Not mentioned	0·1% Bromfenac	31	68 (8)	201 (20)
						0·1% betamethasone	31	69 (10)	203 (23)
Entezari 2017	Iran	DM with and without DR	3 months	Steroid eye drop	Based on OCT	Artificial tear	54	69 (6)	235 (17)
						0·1% diclofenac	54	67 (8)	239 (16)
Fard 2011	Iran	DM with moderate-severe NPDR	6 months	Not mentioned	Based on OCT	Sham	30	60 (4)	170 (28)
						IV-B (1·25 mg)	31	62 (5)	169 (25)
Howaidy 2021	Egypt	T2D with BDR or mild NPDR	3 months	1% Prednisolone	Based on OCT	None	41	62 (5)	268 (12)
						0·1% Nepafenac	38	63 (4)	267 (14)
						IV-R (0·5 mg)	37	65 (3)	269 (16)
Khodabandeh 2018	Iran	T2D with BDR or mild NPDR	3 months	Not mentioned	CST >300 μm	None	35	66 (11)	268 (27)
						IV-B (1·25 mg)	36	62 (6)	261 (24)
Kim 2008	South Korea	T2D with mild or moderate NPDR	6 months	1% prednisolone	Based on OCT	None	23	67 (10)	205 (39)
						Sub-tenon TA	23	68 (10)	228 (43)
Mokbel 2019	Egypt	DM without DR	3 months	Steroid eye drop	CST changes >30 μm	None	50	54 (8)	216 (25)
						0·1% Nepafenac	50	55 (7)	220 (22)
Pollack 2017	USA, Europe, Middle East, and Asia	DM with NPDR	3 months	0·1% dexamethasone	CST changes >30% increase	None	80	69 (8)	277 (23)
						0·1% Nepafenac	80	68 (9)	269 (29)
Sarfraz 2017	Pakistan	DM with NPDR	3 months	1% prednisolone	CST changes >10% increase	None	30	61 (5)	224 (12)
						0·1% Nepafenac	30	61 (5)	226 (11)
Singh 2012	U.S.A	DM with NPDR	3 months	1% prednisolone	CST changes >30% increase	None	126	66 (10)	204 (25)
						0·1% Nepafenac	125	67 (10)	198 (27)
Singh 2017	U.S.A, Latin America, and the Caribbean	DM with NPDR	3 months	1% prednisolone	CST changes >30% increase	None	593	67 (8)	248 (24)
						0·3% Nepafenac	587	67 (9)	246 (25)
Song 2020	U.S.A	DM with NPDR or inactive PDR	3 months	1% prednisolone	CST changes >30% increase	Sham	15	66 (NA)	251 (NA)
						IV-A (2 mg)	15	66 (NA)	263 (NA)
Udaondo 2011	Spain	DM with NPDR	3 months	0.1% dexamethasone	Based on OCT	Sham	27	69 (5)	202 (7)
						IV-R (0·5 mg)	27	73 (5)	198 (NA)

BDR, background diabetic retinopathy; CST, central subfield thickness; FA, fluorescein angiography; IV-TA, intravitreal Triamcinolone Acetonide injection; IV-R, intravitreal Ranibizumab injection; IV-A, intravitreal Aflibercept injection; IV-B, intravitreal Bevacizumab injection; Sub-tenon TA, Sub-tenon injection of Triamcinolone Acetonide; NPDR, non-proliferate diabetic retinopathy; OCT, optical coherence tomography; PDR, proliferate diabetic retinopathy; T2D, type 2 diabetes;.

The quality of the included trials is shown in Supplementary Figure 1. Overall, the trials included in this study exhibited a low to moderate risk of bias. However, participants and outcome assessors in most studies could not be masked because of the physical nature of treatments (eye drop, intravitreal injection). Unmasked participants and outcome assessors might increase the risk of bias.

### PME outcomes

**Figure 3:**
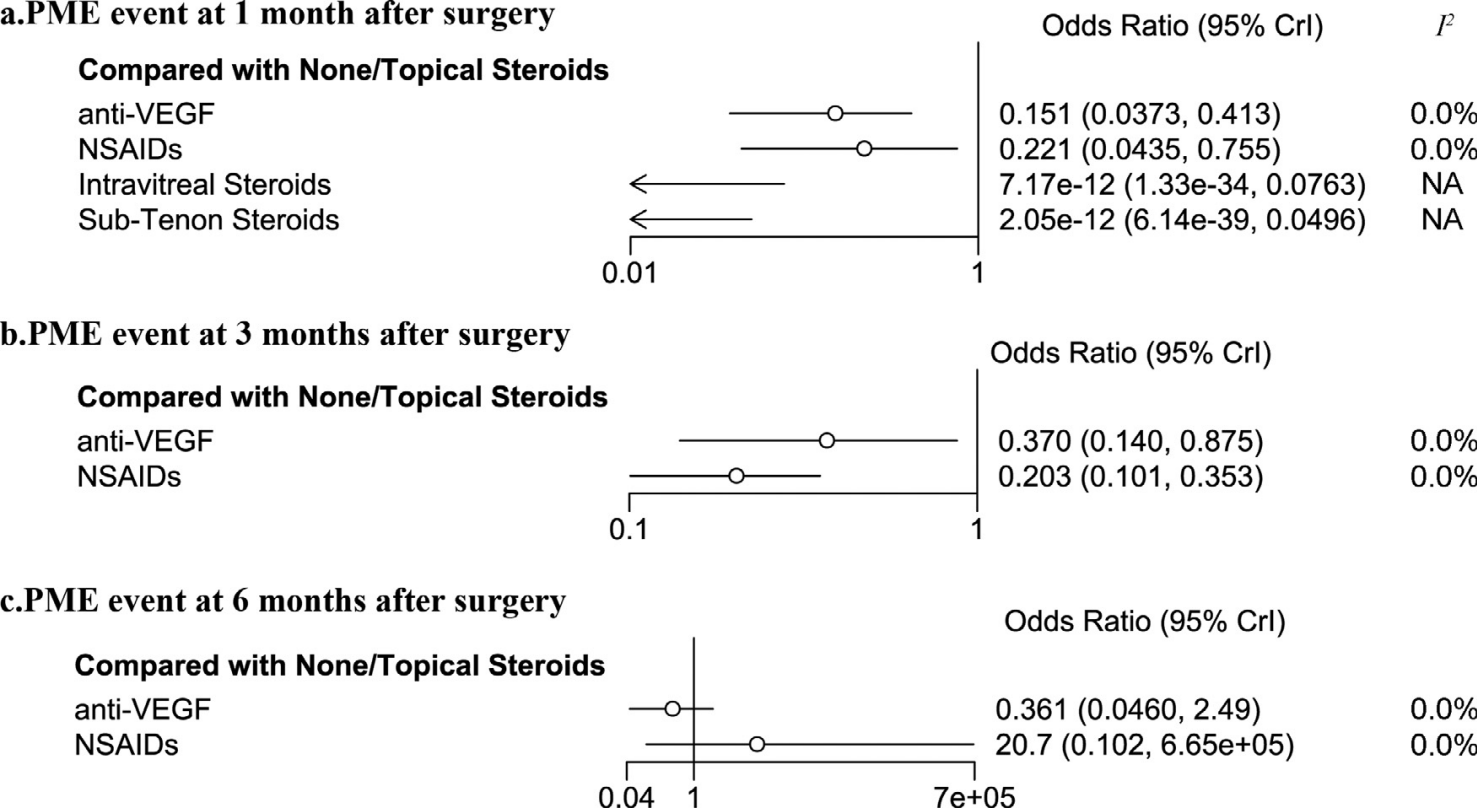
Forest plot of postoperative macular edema outcome in diabetes patients after cataract surgery. The forest plots exhibited the effect of different prophylactic interventions for preventing postoperative macular edema (PME) at 1 month (a), 3 months (b), and 6 months (c) after cataract surgery. Each horizontal line on forest plots represents the pooled odds ratio of individual intervention (compared with None/Topical Steroids alone), with the odds ratio plotted as a circle and the 95% confidence interval plotted as the line. Clip confidence intervals to arrows when they exceed specified limits. When the odds ratio is less than 1, the specified intervention is associated with lower risk of PME than None/Topical Steroids alone. anti-VEGF, intravitreal anti-vascular endothelial growth factor injection; NSAIDs, Nonsteroidal anti-inflammatory drugs; *I^2^*, heterogeneity; 95% Crl, 95% confidence interval. NA, not applicable because of insufficient direct comparisons for calculation.

These are insufficient PME events in 1 week after surgery. At 1 month after surgery, patients received additional topical NSAIDs (OR=0·221, 95%CI: 0·044–0·755, *I^2^*=0·0%, 5 studies), intravitreal anti-VEGF agents (OR=0·151, 95%CI: 0·03–0·413, *I^2^*=0·0%, 5 studies) injection, intravitreal steroid injection, and sub-tenon steroid injection exhibited a significant lower risk of PME, compared to None/Topical Steroid. At 3 months after surgery, both additional topical NSAIDs (OR=0·370, 95%CI: 0·140–0·875, 8 studies) and intravitreal anti-VEGF agents (OR=0·203, 95%CI: 0·101–0·353, *I^2^*=0·0%, 4 studies) are significantly effective in preventing PME. Such protective effect did not reach statistical significance at 6 months after surgery (Figure 3, and Supplementary Figure 2).

Only one study provides evidence regarding the indirect comparison between None/Topical Steroids and other 2 interventions (anti-VEGF and NSAIDs). Thus, there was no evidence of statistically significant inconsistency for all PME outcomes. Comparison-adjusted funnel plot was used to assess publication bias for PME outcome at 1 month and 3 months after surgery and suggested no evidence of publication bias or small-study effects (Supplementary Figure 3–4).

One included study described PME outcome at 3 months after surgery was classified as high risk of bias. Sensitive analysis revealed that excluding this study did not influence the results (**Supplementary Figure 5**).

### BCVA outcomes

**Figure 4:**
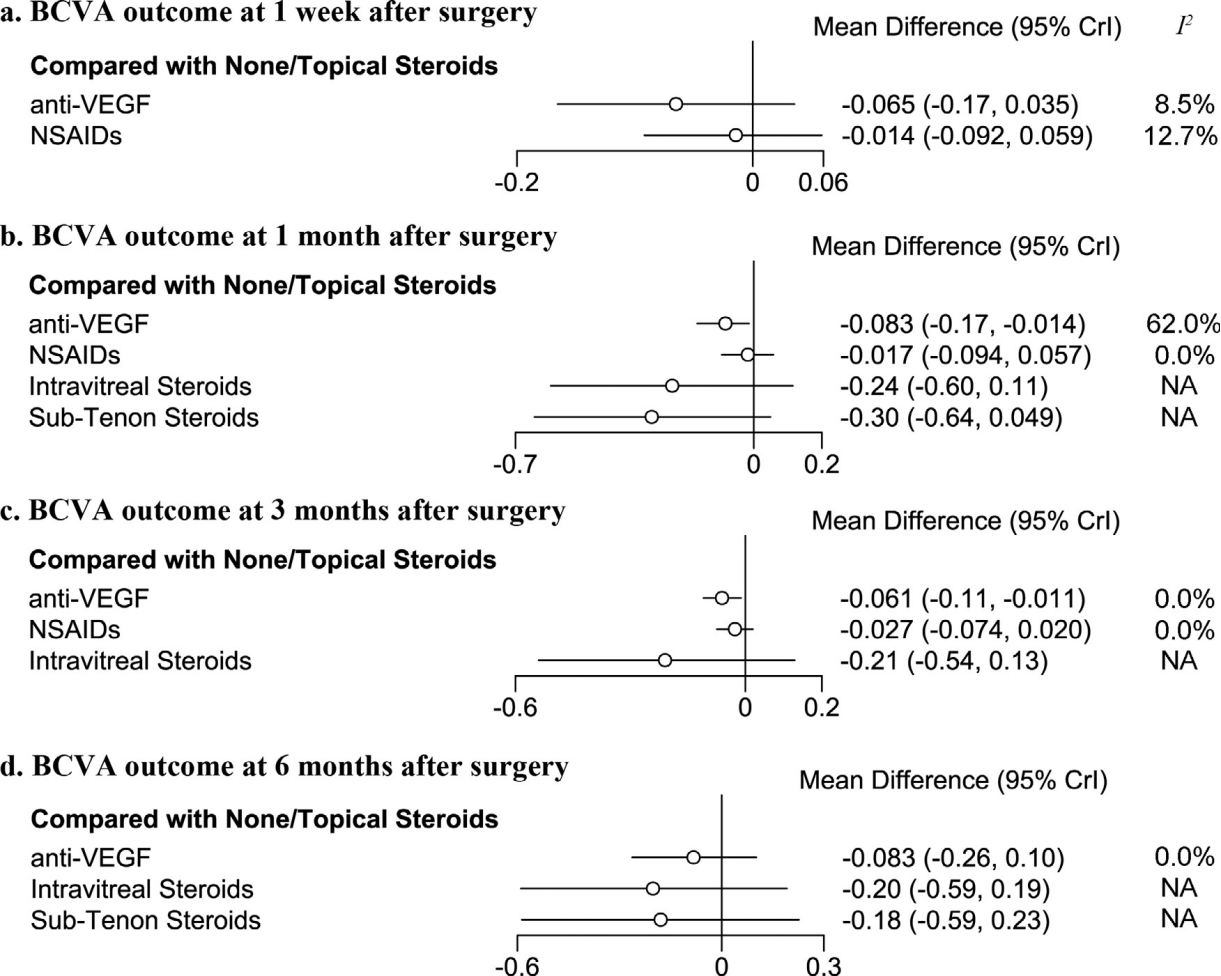
Forest plot of best-corrected visual acuity outcome in diabetes patients after cataract surgery. The forest plots exhibited the effect of different prophylactic interventions for improving postoperative BCVA at 1 week (a), 1 month (b), 3 months (c), and 6 months (d) after cataract surgery. Best-corrected visual acuity (BCVA) is expressed as Logarithm of the Minimum Angle of Resolution (LogMAR). Each horizontal line on forest plots represents the pooled mean difference of individual intervention (compared with None/Topical Steroids alone), with the mean difference plotted as a circle and the 95% confidence interval plotted as the line. When the effect size is less than 0, the specified treatment is associated with better BCVA outcome than None/Topical Steroids alone. anti-VEGF, intravitreal anti-vascular endothelial growth factor injection; NSAIDs, Nonsteroidal anti-inflammatory drugs; *I^2^*, heterogeneity; 95% Crl, 95% confidence interval. NA, not applicable because of insufficient direct comparisons for calculation.

BCVA was recorded and transformed into LogMAR units. At 1 week after surgery, neither additional topical NSAIDs (MD=−0·065, 95%CI: −0·17 to 0·035, *I^2^*=8·5%, 2 studies) nor intravitreal anti-VEGF agents (MD=−0·014, 95%CI: −0·092 to 0·059, *I^2^*=12·7%, 3 studies) showed superior BCVA outcome, compared to None/Topical Steroids. Surprisingly, only additional anti-VEGF exhibited better BCVA outcome at 1 month and 3 months after cataract surgery in diabetes patients. Compared to None/Topical Steroids, patients perioperatively treated with additional anti-VEGF have −0·083 MD (95%CI: −0·17 to −0·014, *I^2^*=62·0%, 5 studies) of LogMAR at 1 month after surgery, which is equivalent to about 4·2 ETDRS letter. At 3 months, patients treated with additional anti-VEGF have a less magnitude but significant better BCVA than None/Topical Steroids (LogMAR MD=−0·061, 95%CI: −0·11 to −0·011, *I^2^*=0·0%, 5 studies), which is equivalent to about 3·1 ETDRS letter. In contrast, additional NSAIDs did not show superior BCVA outcome than None/Topical Steroids at 1 month (MD=−0·017, 95%CI: −0·094 to 0·057, *I^2^*=0·0%, 5 studies) and 3 months (MD=−0·027, 95%CI: −0·076 to 0·020, *I^2^*=0·0%, 5 studies) after cataract surgery, so did the intravitreal steroids and sub-tenon steroids injection (Figure 4 and Supplementary Figure 6). At 6 months after surgery, no study described the BCVA outcome of diabetes patients who received additional NSAIDs eye drop. All other perioperative interventions did not exhibit additional benefits to BCVA outcome than reference intervention.

Similar to PME outcome, there was no evidence of statistically significant inconsistency for BCVA outcome because of lacking indirect comparison. Comparison-adjusted funnel plot indicated no evidence of publication bias or small-study effects (Supplementary Figure 7–8).

## Discussion

We analyzed 2566 participants from 17 randomized controlled trials in the present Bayesian network meta-analysis. We found that compared to None/Topical Steroids eye drops alone, diabetes patients who received additional anti-VEGF and NSAIDs were less likely to develop PME at 1 and 3 months after cataract surgery. Furthermore, patients who received additional anti-VEGF showed better BCVA outcomes at 1 and 3 months after cataract surgery. At 6 months after surgery, none of these interventions brought additional benefits to anatomic and visual acuity outcomes.

It is essential that the data extracted for a network meta-analysis meet the consistency assumption.^40^ Although we did not find any statistical findings (nodesplit method) of inconsistency for PME and BCVA outcome because of lacking indirect comparison, the presence of inconsistency cannot be fully excluded. The consistency hypothesis is valid when all included studies are “jointly randomizable”, which means that each enrolled subject in a given study would be eligible for enrollment in the other studies.^41^ In the present network meta-analysis, however, DR severity of enrolled patients across 17 studies were not identical (Table 1). Most studies included patients without DR or with mild/moderated NPDR, whereas some studies also included severe NPDR and PDR patients after pan-retinal photocoagulation treatment.^13^^,^^30^^,^^37^^,^^38^ Such different severity of DR fundamentally increases the susceptibility of PME development.^4^ Furthermore, in contrast to prescribing steroid eye drop, about 1/3 included studies did not prescribe or describe any routine perioperative interventions. Statistically, we used both placebo and steroid eye drop as reference intervention, which might inevitably induce inconsistency.

Similar to pair-wise meta-analysis, the reliability of the result also lies on the homogeneity assumption. In current studies, we did not detect significant heterogeneity in PME outcomes. Compared to None/Topical Steroids alone, the effect of additional anti-VEGF on BCVA outcome at 1 month after surgery exhibited high heterogeneity (*I^2^*=62·0%). This heterogeneity mainly originated from an individual study.^31^ In this study, the researchers included moderate and severe NPDR patients and did not prescribe any perioperative interventions in the control group. Mean central macular thickness of the placebo group significantly increased from 170 µm to 260 µm at 1 month after cataract surgery. After excluding this study, we found some evidence that anti-VEGF was superior (MD=−0·057, 95%CI: −0·12 to 0·003, *P* = 0·07, *I^2^*=0·0%, 5 studies, Supplementary Figure 9–10) to None/Topical Steroids on BCVA outcome at 1 month after surgery. Differences between groups did not meet conventional levels of statistical significance. We also explored publication bias through comparison-adjusted funnel plots, and find no evidence for publication bias and small-study effects across all outcomes.

In the present study, we found that additional NSAIDs were superior to None/Topical Steroids alone in short-term PME outcomes. During cataract surgery, intraocular lens implantation induces proinflammatory cytokines production in the lens epithelial cells, including prostaglandins.^42^ Prostaglandins disrupts blood-eye barrier integrity.^43^ Thus, the effects of NSAIDs, prostaglandins synthesize inhibitors, on PME prevention has been well-documented in nondiabetic and diabetic populations.^17^ As PME often occurs in a few weeks to months after cataract surgery,^8^ we did not observe any additional effect of NSAIDs over None/Topical Steroids alone at 1 week after cataract surgery. We also did not observe any long-term protective effect of additional NSAIDs (at 6 months), probably because PME resolves spontaneously in its natural course.^44^ However, the protective effect of additional NSAIDs on anatomic outcome seemed to bring additional improvement in visual acuity outcome. Previously, extensive studies investigated the effect of NSAIDs in cataract surgery but did not find solid evidence of prophylactic NSAIDs on vision acuity outcome among general cataract patients.^45^^,^^46^ In the present study, we also failed to find evidence that additional NSAIDs is superior to None/Topical Steroids alone on BCVA outcome at 1 month (MD=−0·017, 95%CI: −0·094 to 0·057, *I^2^*=0·0%, 5 studies) and 3 months (MD=−0·027, 95%CI: −0·076 to 0·020, *I^2^*=0·0%, 5 studies). Such evidence did not support the combination of topical NSAIDs and steroids as the prophylactic intervention for cataract surgery among diabetes patients.

In all included studies, single dose of anti-VEGF was given at the end of cataract surgery. We found that additional anti-VEGF intervention surpassed None/Topical Steroids alone in preventing PME and improving postoperative BCVA. Traditionally, anti-VEGF is used as the primary therapy for DME.^12^ Recently, anti-VEGF is used to prevent the exacerbation of active DME after cataract surgery.^47^, ^48^, ^49^ The combined anti-VEGF and cataract surgery has significantly improved BCVA of diabetes patients with active DME (LogMAR at 6 months: 0·24 ± 0·27).^47^ In the present study, we found that compared to None/Topical Steroids alone, patients perioperatively treated with additional anti-VEGF have 4·2 and 3·1 ETDRS letters more improvement at 1 month and 3 months after surgery. Such evidence supports the additional anti-VEGF injection as the prophylactic intervention for cataract surgery among diabetes patients. Further investigations are needed to explore the effect of additional anti-VEGF on diabetes patients with different DR severity who underwent cataract surgery. Given that about 10% of these patients develop PME under traditional perioperative interventions,^4^ findings from OCTA and the occurrence of intraoperative complications may further guide the precision application of anti-VEGF.

Using prophylactic intravitreal steroids in diabetic patients undergoing cataract surgery is inconclusive because of a limited data source. There is currently emerging evidence of using intravitreal steroids in cataract surgery among patients with pre-existing DME.^50^ Intravitreal dexamethasone implant (IDI) provides a longer-lasting anti-inflammation effect. A nest case-control study revealed that using IDI at the end of cataract surgery successfully decreased central subfield thickness and improved BCVA outcome.^51^ The timing of performing IDI seems irrelevant to BCVA and anatomic outcome. Delivering IDI 1 month prior or after cataract surgery showed comparable outcomes than concomitant with cataract surgery.^52^^,^^53^

Our studies have several strengths. Through extensive literature searching and the Bayesian network meta-analysis method, we were able to compare treatments that have not been compared in previous head-to-head studies. Furthermore, we reported our findings according to recommendations of PRISMA-NMA statement and did not find evidence of publication bias or inconsistency. Moreover, we first found that additional anti-VEGF intervention was the only kind of intervention that surpassed None/Topical Steroids alone in preventing PME and improving postoperative BCVA among diabetes patients who underwent cataract surgery.

However, some limitations should also be mentioned. First, prophylactic intervention agents vary for each individual studies. For example, different kinds of VEGF agents (Bevacizumab, Ranibizumab and Aflibercept) and NSAIDs agents (Nepafenac, Ketorolac, Bromfenac, and Diclofenac) were used among included studies. Currently, The number of included studies currently limited further subgroup analysis regarding different agents. Second, both placebo and topical steroids are used as controls. Such difference may be the major cause that induced heterogeneity in this study. Third, participants and outcome assessors in most studies could not be masked because of the physical nature of treatments. However, as PME and BCVA were assessed by objective methods, the unmasked intervention probably did not affect outcome evaluation. Last, insufficient data results in low certainty of some treatments, such as intravitreal and sub-tenon steroids.

In conclusion, compared to topical steroid eye drop alone, both additional NSAIDs and anti-VEGF effectively prevent PME among diabetes patients who underwent cataract surgery. For visual acuity outcome, the additional anti-VEGF intervention was associated with better postoperative BCVA. Future studies should be warranted to corroborate our findings.

## Contributors

Conception and design of the research: RHZ, LD and WBW; Acquisition and interpretation of the data: RHZ, DL, WBW and QY, YML, HYL, YTL, CYY; Statistical analysis and writing of the manuscript: RHZ, LD, WDZ, HTW, YFL, and WBW; Critical revision of the manuscript: RHZ and WBW.

## Funding

Capital Health Research and Development of Special (2020-1-2052) and Science & Technology Project of Beijing Municipal Science & Technology Commission (Z201100005520045, Z181100001818003).

### Data sharing statement

**D**ata used this study was collected from previously published literature. Extracted data are available within the paper and its supplementary material.

## Declaration of interests

None.

## Appendix Supplementary materials

Image, application 1

Image, application 2
