# Supplementary material for: Prophylactic interventions for preventing macular edema after cataract surgery in patients with diabetes: A Bayesian network meta-analysis of randomized controlled trials
Source: eClinicalMedicine. 2022 May 20;49:101463. doi: 10.1016/j.eclinm.2022.101463 (PMC9124709; doi:10.1016/j.eclinm.2022.101463)
Supplement: Supplementary file 1 [file mmc1.docx]

**Search strategy**

**Supplementary Figure 1.** The quality of the included trials

**Supplementary Figure 2.** Ranking probabilities of preventing the occurrence of PME in diabetes patients after cataract surgery.

**Supplementary Figure 3.** Comparison adjusted funnel plot for PME outcome at 1 month after surgery.

**Supplementary Figure 4.** Comparison adjusted funnel plot for PME outcome at 3 months after surgery.

**Supplementary Figure 5.** Forest plot of PME outcome at 3 months after cataract surgery (Fard 2011 excluded)

**Supplementary Figure 6.** Ranking probabilities of preventing the occurrence of BCVA in diabetes patients after cataract surgery.

BCVA is expressed as LogMAR

**Supplementary Figure 7.** Comparison adjusted funnel plot for BCVA outcome at 1 month after surgery.

**Supplementary Figure 8.** Comparison adjusted funnel plot for BCVA outcome at 3 months after surgery.

**Supplementary Figure 9.** Forest plot of BCVA outcome in diabetes patients at 1 month after cataract surgery (Fard 2011 excluded)

**Supplementary Figure 10.** Comparison adjusted funnel plot of BCVA outcome in diabetes patients at 1 month after cataract surgery (Fard 2011 excluded)

**Supplementary Table 1.** Included studies in each endpoint.
