# Supplementary material for: Prophylactic interventions for preventing macular edema after cataract surgery in patients with diabetes: A Bayesian network meta-analysis of randomized controlled trials
Source: eClinicalMedicine. 2022 May 20;49:101463. doi: 10.1016/j.eclinm.2022.101463 (PMC9124709; doi:10.1016/j.eclinm.2022.101463)
Supplement: Supplementary file 2 [file mmc2.docx]

**Search strategy**

**MEDLINE(Ovid) and Embase(Ovid):** exp macular edema cystoid/ or exp macula lutea/ or (macula$ ADJ3 oedema).tw. or (macula$ ADJ3 edema).tw. or (CME or CMO).tw. or (Macular Edema).tw. or (Macular oedema).tw.) AND (exp Diabetes Mellitus/ or (diabetic).tw. or (diabetic retinopathy).tw.) AND (exp Cataract/ or exp Cataract Extraction/ or exp Phacoemulsification/ or (cataract ADJ3 (surg* or extract*)).mp. or (cataract* or sutureless or nonstitch* or non stitch* or no stitch* or nostitch or phako* or phaco*).ti,ab.

**Web of Science:** (macular edema cystoid or Macular Edema or Macular oedema) AND (Diabetes Mellitus or diabetic or diabetic retinopathy) AND (Cataract or Phacoemulsification)

**ClinicalTrials:** diabetes and cataract and macular edema

**Supplementary Figure 1.** The quality of the included trials

**Supplementary Figure 2.** Ranking probabilities of preventing the occurrence of PME in diabetes patients after cataract surgery.


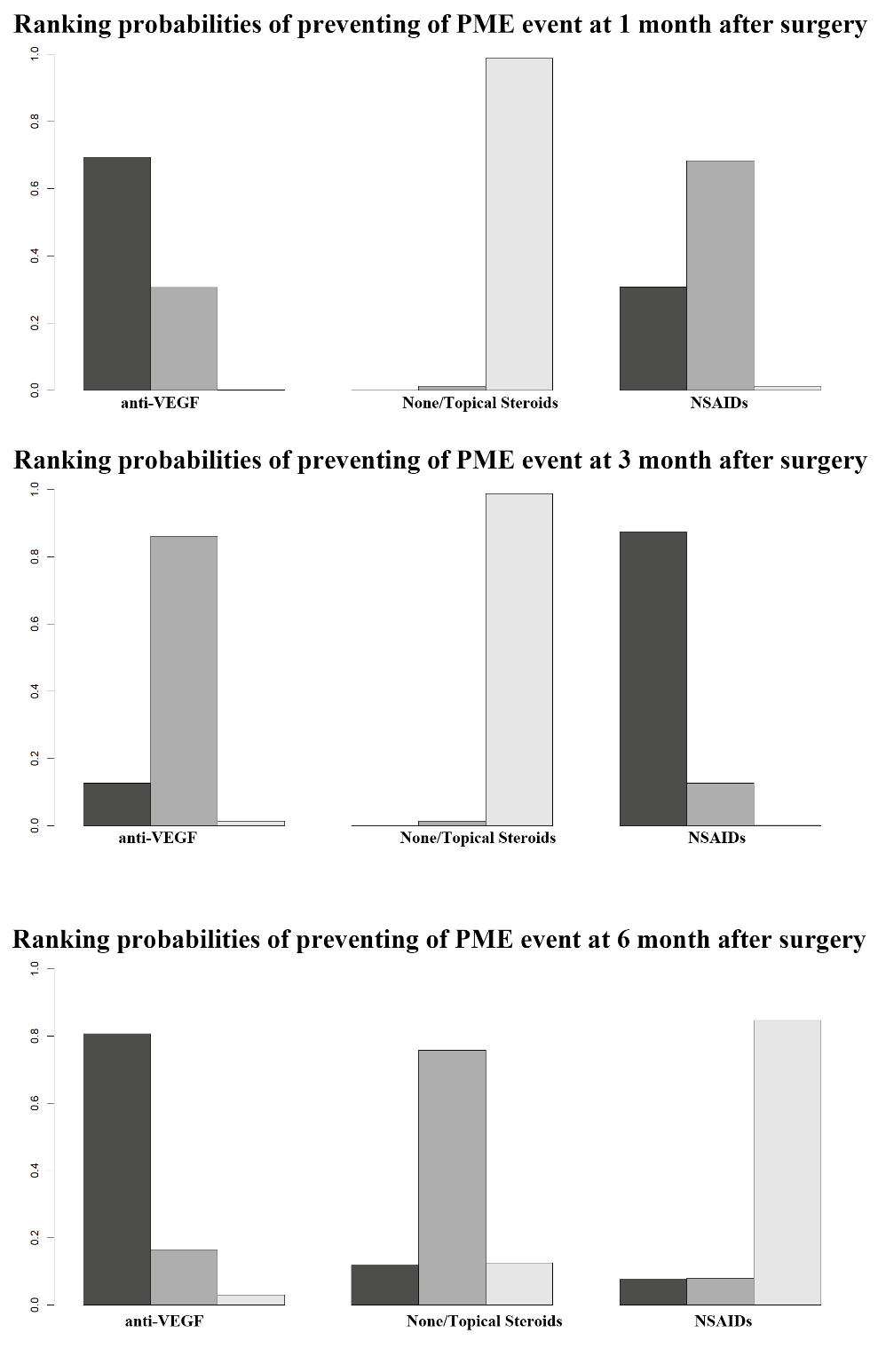


**Supplementary Figure 3.** Comparison adjusted funnel plot for PME outcome at 1 month after surgery.


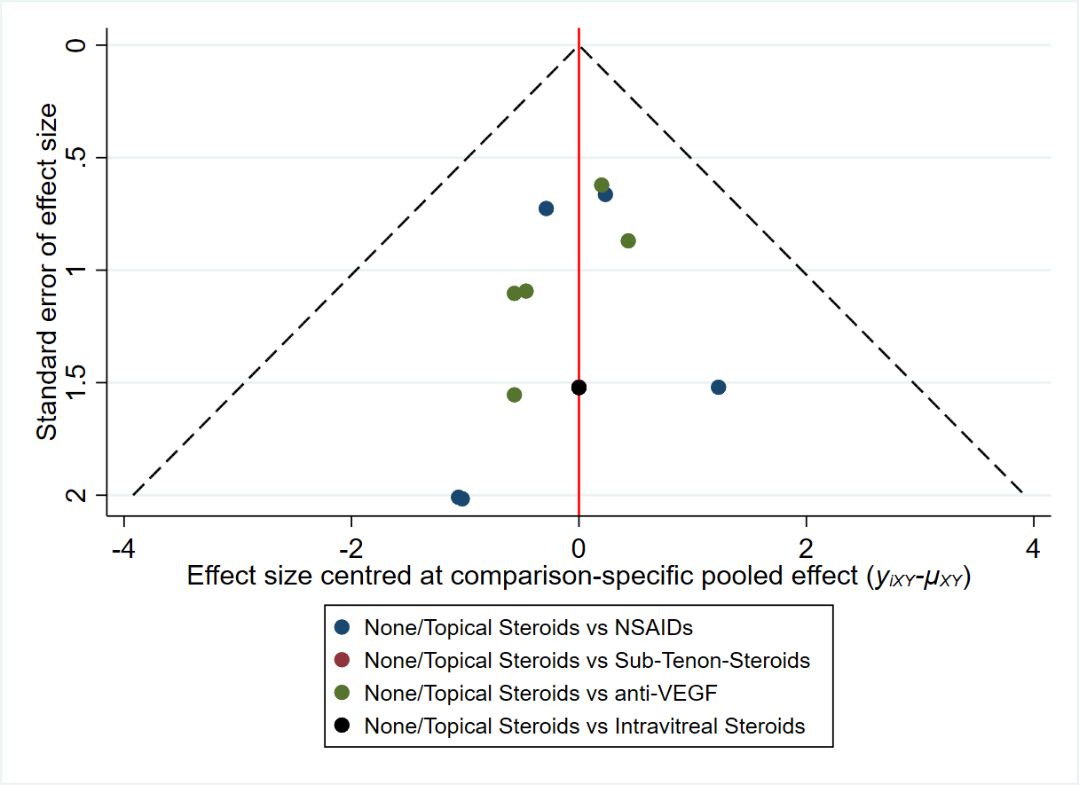


**Supplementary Figure 4.** Comparison adjusted funnel plot for PME outcome at 3 months after surgery.


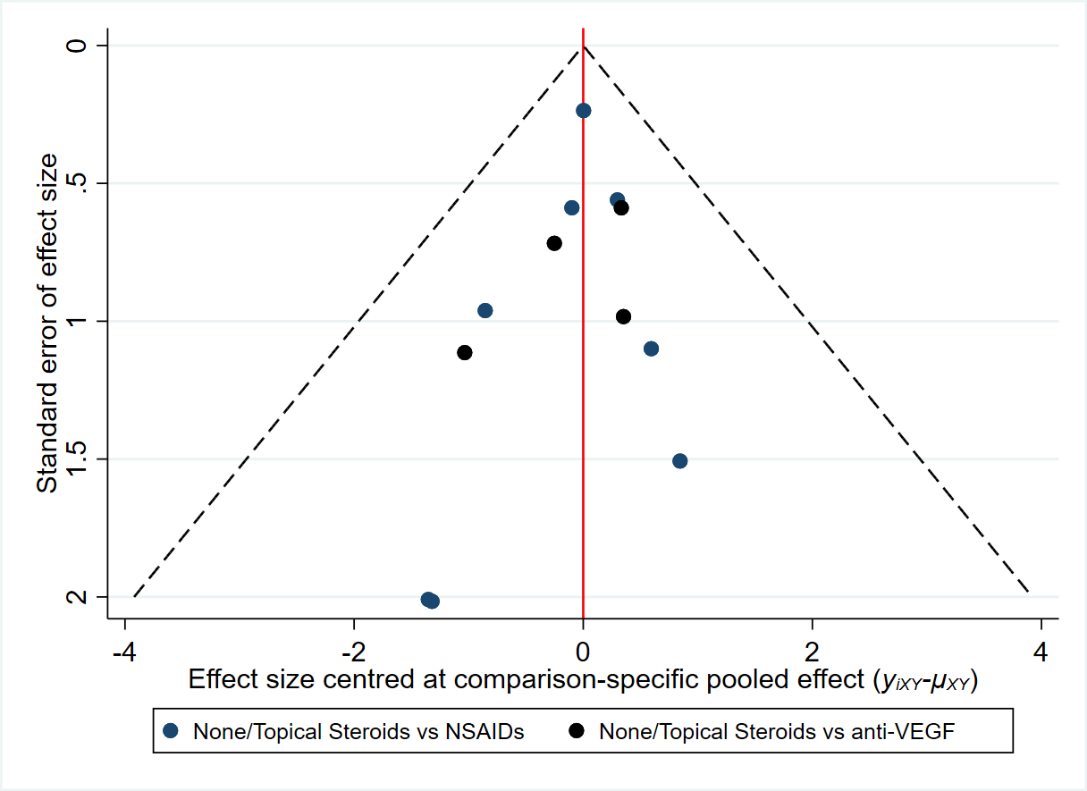


**Supplementary Figure 5.** Forest plot of PME outcome at 3 months after cataract surgery (one study with high risk of bias excluded)


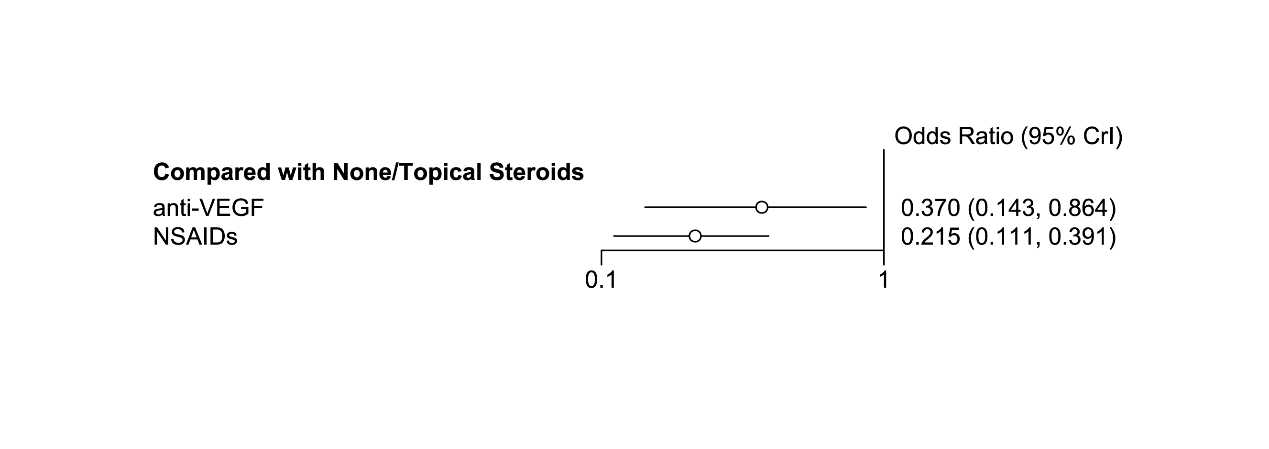


**Supplementary Figure 6.** Ranking probabilities of preventing the occurrence of BCVA in diabetes patients after cataract surgery.


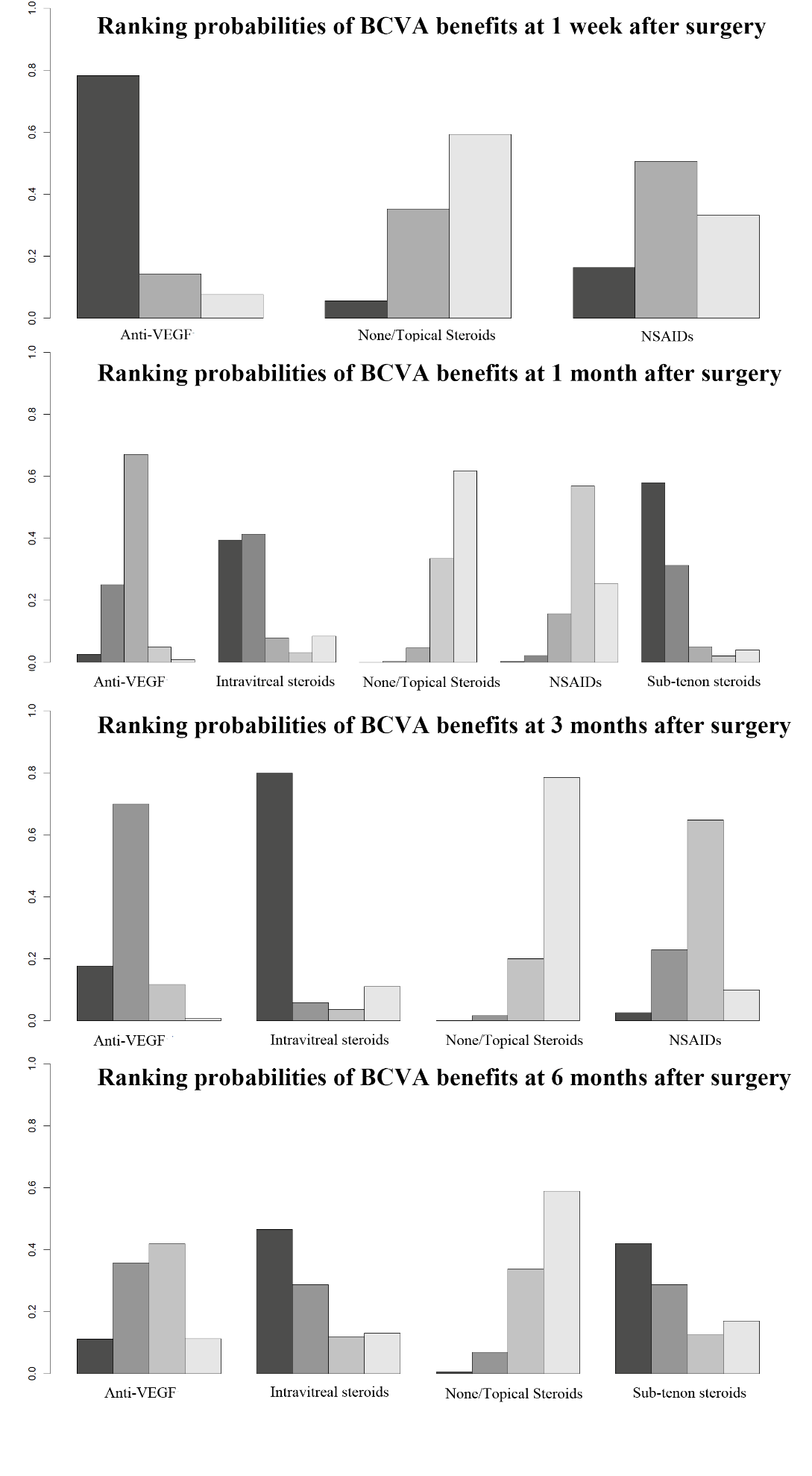


BCVA is expressed as LogMAR

**Supplementary Figure 7.** Comparison adjusted funnel plot for BCVA outcome at 1 month after surgery.


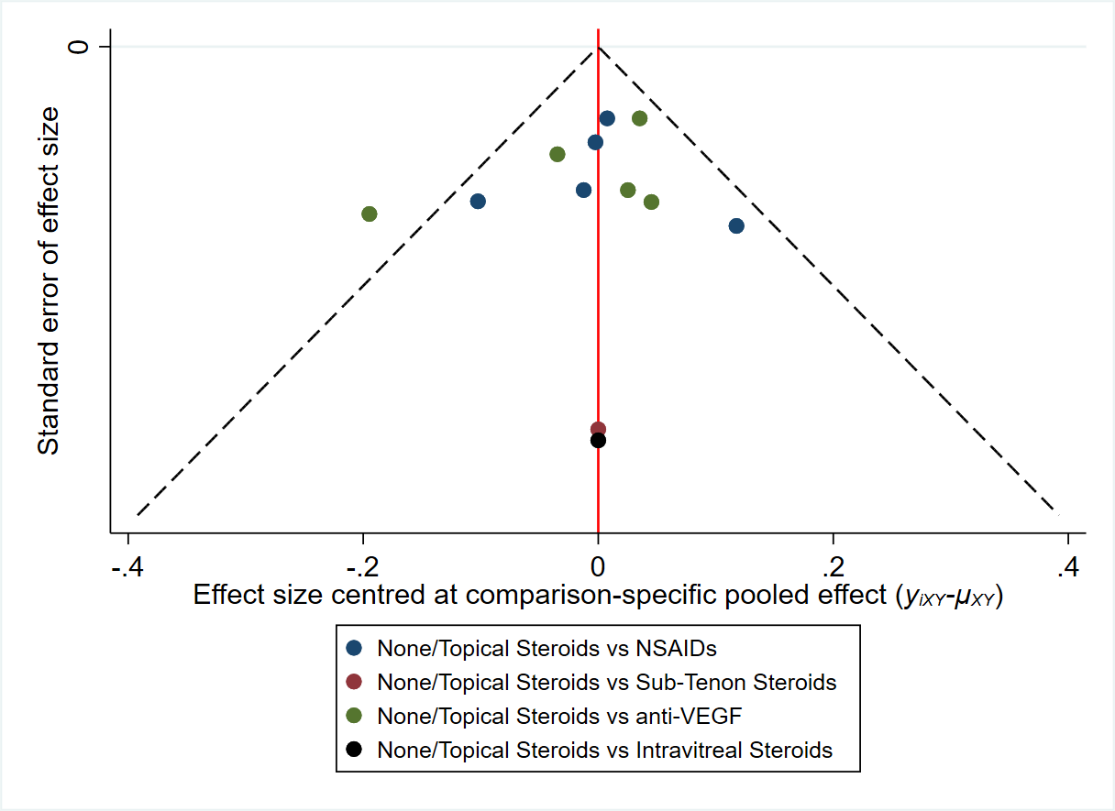


**Supplementary Figure 8.** Comparison adjusted funnel plot for BCVA outcome at 3 months after surgery.


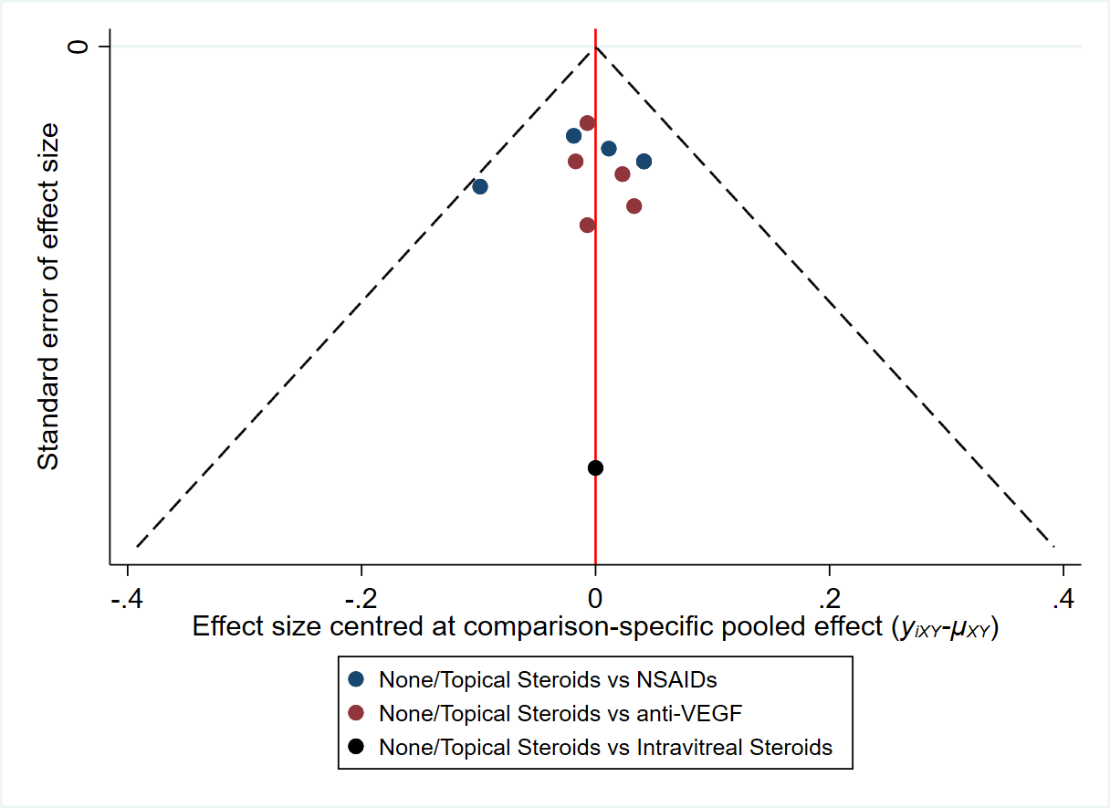


**Supplementary Figure 9.** Forest plot of BCVA outcome in diabetes patients at 1 month after cataract surgery (Fard 2011 excluded)


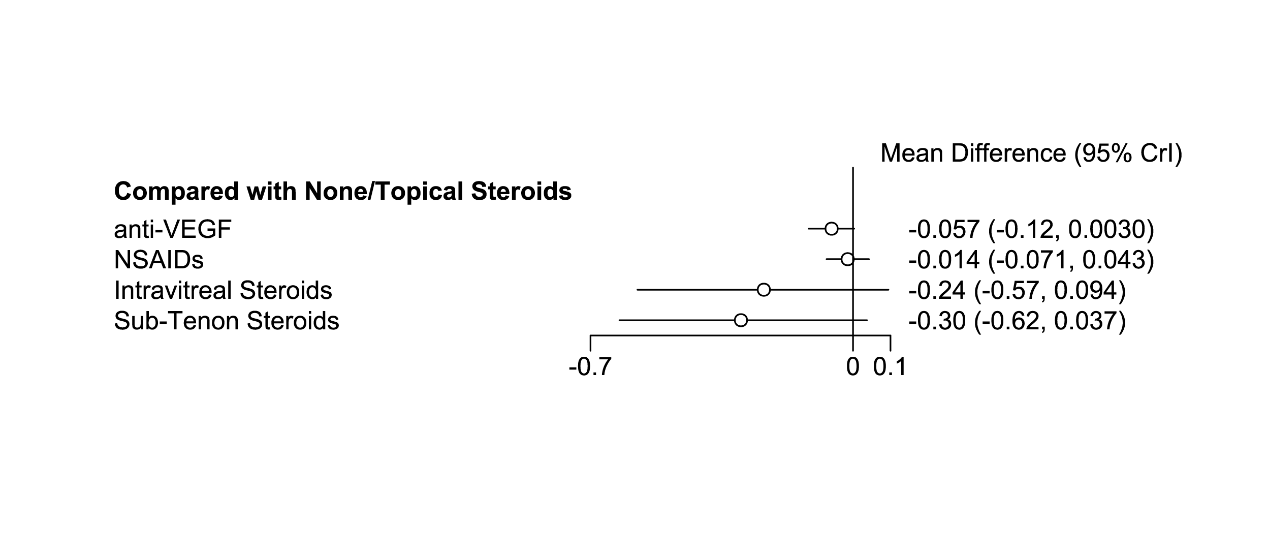
 **Supplementary Figure 10.** Comparison adjusted funnel plot of BCVA outcome in diabetes patients at 1 month after cataract surgery (Fard 2011 excluded)


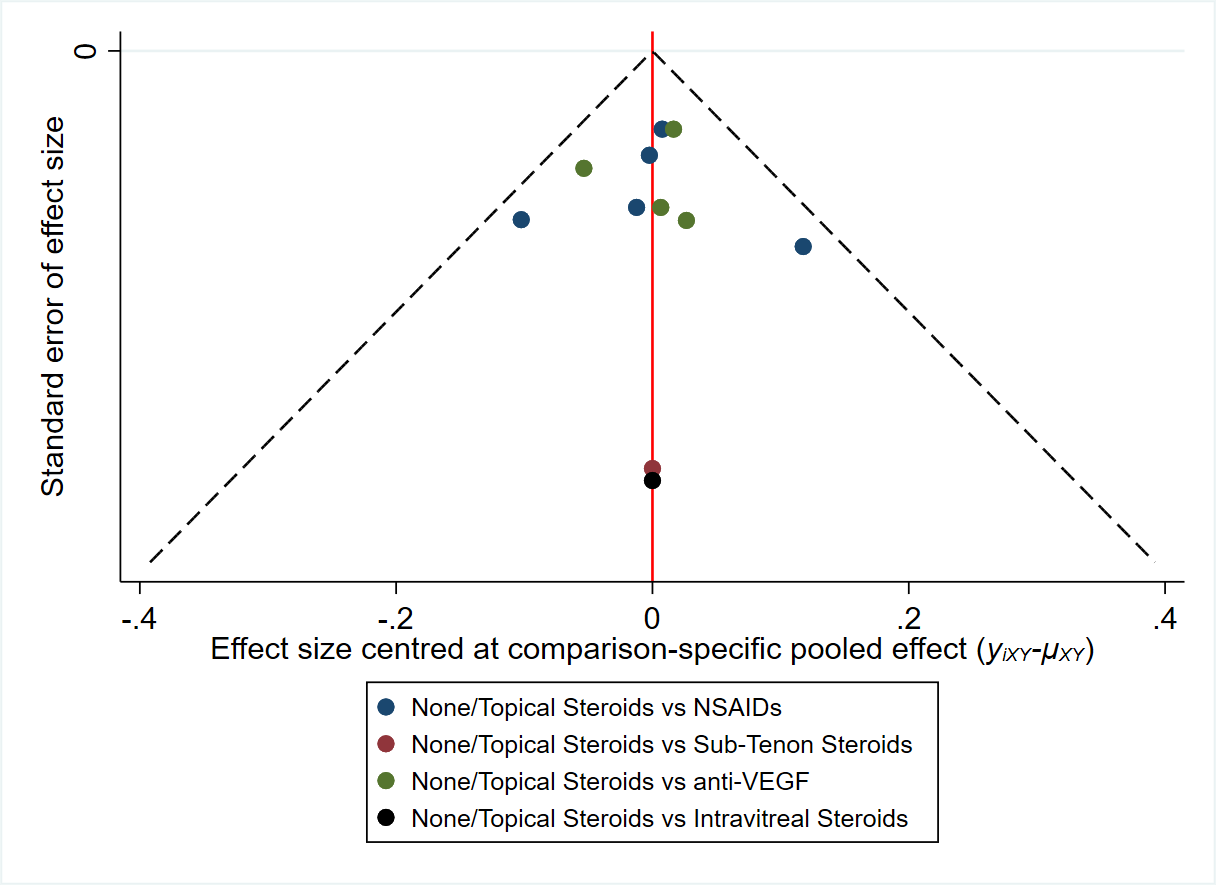


Supplementary Table 1. Included studies in each endpoint.

|  | Postoperative macular edema | | | Best-corrected visual acuity | | | |
| --- | --- | --- | --- | --- | --- | --- | --- |
|  | 1 month | 3 months | 6 months | 1 week | 1 month | 3 months | 6 months |
| Ahmadabadi 2010 | √ |  |  |  | √ | √ | √ |
| Alnagdy 2018 |  | √ |  |  |  |  |  |
| Chae 2014 | √ | √ | √ | √ | √ | √ | √ |
| Elsawy 2013 | √ | √ | √ |  |  |  |  |
| Endo 2010 |  |  |  |  | √ |  |  |
| Entezari 2017 | √ | √ | √ |  | √ | √ |  |
| Fard 2011 | √ | √ | √ |  | √ | √ | √ |
| Howaidy 2021 | √ |  |  | √ | √ | √ |  |
| Khodabandeh 2018 | √ | √ |  |  | √ | √ |  |
| Kim 2008 | √ |  |  |  | √ |  | √ |
| Mokbel 2019 |  |  |  | √ | √ | √ |  |
| Pollack 2017 |  | √ |  |  |  | √ |  |
| Sarfraz 2017 |  | √ |  |  |  |  |  |
| Singh 2012 |  | √ |  | √ | √ | √ |  |
| Singh 2017 |  | √ |  |  |  |  |  |
| Song 2020 | √ | √ |  |  | √ | √ |  |
| Udaondo 2011 | √ | √ |  |  |  |  |  |
